# Combatting Sedentary Behaviors by Delivering Remote Physical Exercise in Children and Adolescents with Obesity in the COVID-19 Era: A Narrative Review

**DOI:** 10.3390/nu13124459

**Published:** 2021-12-14

**Authors:** Matteo Vandoni, Roberto Codella, Roberto Pippi, Vittoria Carnevale Pellino, Nicola Lovecchio, Luca Marin, Dario Silvestri, Alessandro Gatti, Vittoria Carlotta Magenes, Corrado Regalbuto, Valentina Fabiano, Gianvincenzo Zuccotti, Valeria Calcaterra

**Affiliations:** 1 Laboratory of Adapted Motor Activity (LAMA), Department of Public Health, Experimental Medicine and Forensic Science, University of Pavia, 27100 Pavia, Italy; vittoria.carnevalepellino@unipv.it (V.C.P.); alessandro.gatti08@universitadipavia.it (A.G.); 2 Department of Endocrinology, Nutrition and Metabolic Diseases, IRCCS MultiMedica, 20138 Milan, Italy; roberto.codella@unimi.it; 3 Department of Biomedical Sciences for Health, Università degli Studi di Milano, 20133 Milan, Italy; 4 Healthy Lifestyle Institute, C.U.R.I.A.Mo. (Centro Universitario Ricerca Interdipartimentale Attività Motoria), Department of Medicine and Surgery, University of Perugia, 06126 Perugia, Italy; roberto.pippi@unipg.it; 5 Department of Industrial Engineering, University of Rome Tor Vergata, 00133 Rome, Italy; 6 Department of Human and Social Science, University of Bergamo, 24127 Bergamo, Italy; nicola.lovecchio@unibg.it; 7 Laboratory for Rehabilitation Medicine and Sport (LARMS), 00133 Rome, Italy; luca.marin@unipv.it; 8 Department of Research, ASOMI College of Sciences, 2080 Marsa, Malta; direttore@asomi-osteopatia.com; 9 Pediatric Department, “Vittore Buzzi” Children’s Hospital, 20154 Milan, Italy; vittoria.magenes@unimi.it (V.C.M.); valentina.fabiano@unimi.it (V.F.); gianvincenzo.zuccotti@unimi.it (G.Z.); valeria.calcaterra@unipv.it (V.C.); 10 Pediatric and Adolescent Unit, Department of Internal Medicine, University of Pavia, 27100 Pavia, Italy; corrado.regalbuto01@universitadipavia.it; 11 Department of Biomedical and Clinical Science “L. Sacco”, University of Milan, 20157 Milan, Italy

**Keywords:** children obesity, COVID-19, exercise, sedentary, online training program, telehealth, pediatrics

## Abstract

The coexistence of childhood obesity (or its risk) and COVID-19 pandemic put children and adolescents in greater risk to develop respiratory and cardiovascular diseases. In fact, the restrictions introduced to limit the spread of the virus had detrimental effects on various lifestyle components, especially in young population. This resulted in augmented levels of physical inactivity and sedentary behaviors and a reduced time spent in play outdoors or sport practices. Contrariwise, the increased use of technology led clinicians, teachers, and trainers to maintain relations with obese children/adolescents so as to reduce sedentary behaviors and the associated health risks. This narrative review aims to describe the role of Telehealth and Tele-exercise as useful tools in the management of pediatric obesity during COVID-19 pandemic. Telehealth and Tele-exercise were effective in promoting self-monitoring and behavioral changes, including adherence to exercise training programs in children and adolescents. Moreover, tele-exercise platforms such as applications or exergames allowed flexible scheduling, limiting the infection risks.

## 1. Introduction

In response to COVID-19, special measures were implemented by the authorities in order to lower virus spread and reduce the pressure on health systems. School closures and home confinement restrictions changed children’s and adolescents’ everyday routines, modifying their physical activity (PA) and increasing risk of weight gain and obesity development [1,2].

During quarantine, inappropriate computer uses and sedentary occupations have also increased, along with less control by parents over normal practice and healthy behaviors, especially in children at risk for weight gain or already obese/overweight [3].

To limit the spread of the virus, schools and sports facilities, gyms and swimming pools were closed. The possibility to practice sports and to maintain an active lifestyle decreased in children and adolescents with an augmented risk of developing acute and long-term health impairments [4]. In general, children and adolescents with obesity tend to have lower PA levels caused by higher difficulties to perform motor tasks and with fewer possibilities to practice supervised exercise and to interact with peers [5]. Usually, to overcome the difficulties related to exercise practice, in children with fragile conditions, the use of technology through online resources helped the clinicians and trainers to offer training options remotely [6]. Obese and overweight children are already at increased cardiovascular risk; therefore, physical inactivity, which has various other negative effects on their health, must be seriously considered. The negative impact of reduced PA on pediatric obese subjects will depend on the duration of restriction measures. In addition, these restrictions will negatively impact on these subjects’ mental health as well.

The use of telehealth technology is a relatively new approach to deliver healthcare assistance during the outbreak of COVID-19. Telehealth has the potential to promote online exercise or training program so to reduce sedentary behaviors (SB) in children and adolescents [7]. In fact, in the last years, the development of electronic devices and the augmented diffusion of online technologies and training and exercise practice were improved through applications, web channels, and online platforms. In particular, active videogames—exergames—were developed to optimize exercise training with online technologies [8,9].

We are aware of the crucial role of nutrition and lifestyle in weight management, as precedingly addressed on children with obesity during the COVID-19 era [6,10]. Nevertheless, there are few studies resuming the role of online exercise to maintain active and healthy lifestyle in children with obesity. For this reason, at this time, we focused on the possible health gains originating from PA and exercise promotion. In this narrative review, we revised the role of telehealth as an exploitable intervention to remotely deliver physical exercise and to reduce sedentariness in children and adolescents with obesity during COVID-19 era. Considering the impact of the COVID-19 restrictions on the long-term effects on both the physical and the mental health of obese and overweight children, new strategies are mandatory to avoid the obesity-related comorbidities.

## 2. Materials and Methods

This narrative review concerns the influence of the COVID-19 restrictions in PA and SB changes in children and adolescents, focusing on the opportunity of telehealth as a tool to promote online exercise training programs for pediatric patients with obesity. The authors R.P., V.C.P., N.L, D.S., A.G., V.C.M. and C.R. independently identified the most relevant papers published in English in the past 15 years, including original papers, metanalysis, clinical trials, and reviews. Case reports or series and letters were excluded. Papers published up to September 2021 were found thanks to the following keywords: COVID-19, adolescents, children, PA, exercise, training, online, exergames, obesity, lockdown, and weight gain. PubMed, Scopus, EMBASE, and Web of Science were used as the electronic databases to search. Research articles were explained in detail if they full-filled the following criteria: studies with full-text investigating online training or exercise or exergaming in children and adolescents with and without and pre- and during the COVID-19 pandemic. We excluded all the articles that investigated children and adolescents under quarantine or ongoing COVID-19 infectious. The contributions were critically reviewed by M.V., R.C., L.M., V.F. and V.C. and collected by M.V., R.C., R.P., V.C.P., N.L, A.G., V.C.M. and C.R. The final version was approved by all the authors.

## 3. Obesity and Physical Activity

Childhood obesity is a global health issue with a considerable growth in prevalence in the last 25 years, North America and Europe showed the highest prevalence of overweight/obese children in 2006 (~20–30%) [11,12,13,14,15].

Since 2006, the prevalence has almost doubled or tripled, and nearly 170 million children globally are estimated to be overweight or obese.

The proportion of children diagnosed with excess weight (overweight or obese) is becoming higher most rapidly in countries with a good socioeconomic status, although the proportion of overweight and obese children from low- and middle-income areas such as Chile, Egypt, Brazil, and others is reaching similar levels [16].

This emergent and prominent worldwide problem requires attention and appropriate interventions to avoid health-related long-term consequences.

Childhood obesity is known to be associated with increased risk for chronic pathologies like type 2 diabetes, metabolic syndrome, hypertension, cardiovascular disease, stroke, osteoarthritis, mental health issues, and some types of cancer [17]. The increasing prevalence of excess weight in pediatric subjects could also lower the age of onset and the incidence of insulin resistance and cardiovascular alterations [18,19,20].

PA, along with other factors like diet and socioeconomic status, is one of the most important factors to control body weight in pediatric subjects [21].

A reliable PA assessment can be difficult in overweight and obese subjects because of the biomechanics and psychosocial difficulties related to wearing monitoring devices [22,23].

SB and PA should be considered together when examining pediatric subjects’ health [24]. Any waking behavior characterized by an energy consumption ≤1.5 metabolic equivalents while in a sitting, reclining, or lying position defines sedentary behavior [25,26]. Common sedentary habits include smartphone, computer and tablet use, TV viewing, video games playing, driving or riding in a car, and reading while sitting. Excessive sedentary time is globally pervasive among pediatric subjects, and there is a rising evidence on the detrimental effects related to its high levels [27,28].

As reported in Table 1, in subjects up to 2 years of age, the diagnosis of excess weight, either overweightness or obesity, is based on the weight-to-length ratio, using the reference curves of the World Health Organization (WHO). Thereafter, the key diagnostic criterium is the Body Mass Index (BMI) [29].

As stated by the consensus position statement of the Italian Society for Pediatric Endocrinology and Diabetology (SIEDP) and the Italian Society of Pediatrics, it is mandatory to progressively reduce the BMI, especially through changes in eating habits and lifestyle rather than reaching a rapid weight loss through low-calorie diets [30].

It is also necessary to maintain a proper growth rate and achieve a stable weight-to-height ratio and to reduce weight excess, particularly fat mass, trying to preserve the lean mass. Another essential objective is to maintain good mental health (self-esteem, correct attitudes, and health-related life quality).

Several countries (Canada, Australia, USA, UK, and Italy) have adopted the 2020 WHO PA guidelines [31,32,33,34,35]. Children should perform at least an average of 60 min per day (across the week) of “moderate-to-vigorous physical activity” (MVPA), with aerobic activities at a vigorous intensity at least 3 days a week. Doing any physical activities (proper for children’s ability and age) is better than none. Children should gradually increase their frequency, duration, and intensity over time.

Despite all the salutary effects of PA, globally, children were more active decades ago than today. This decrease in PA could be a fundamental contributor to the emerging pandemic of childhood obesity [36].

Achieving the recommended daily MVPA (at least 60 min) is advocated to obtain health benefits. In addition, peer-to-peer interactions during sports (either amateur or agonist) or PA and free play at parks and playgrounds are essential for children’s development of social relationships.

It is well-known that healthy lifestyles and behaviors enhance the immune system [37], reducing the risk for infections and inflammation, and are efficacious in the prevention of various chronic diseases.

However, even during the non-COVID era, more than 80% of children around the world were inactive, nearly 60% did not meet screen time guidelines, and almost a half of them were not compliant to the Mediterranean diet [38,39].

Various studies, mainly concerning football training, showed that regular sports participation might be as effective as common PA involvements in order to improve the body composition, to decrease metabolic alterations, and to improve their psychological states in obese and overweight children [5,40,41].

A commonly examined explanation for the decline in PA is a low amount of MVPA [42,43].

In particular, a decline in regular PA, including active transport, has been noted [44,45].

Sports involvement should be recognized as an effective approach to diminish overweightness and increase PA in children. The WHO also supports the use of the current settings adapted to the national scenarios and cultural attitudes to prevent overweightness and obesity [46].

It is well-known that obesity triggers a systemic inflammatory process originated by a regulatory hormonal dysregulation along with the release of proinflammatory cytokines, even during PA [47]. Nevertheless, various studies show that regular PA is associated with the reversal of the systemic inflammatory condition seen in obese and overweight subjects [48,49]. PA has positive effects on the immune system, and it is considered an “immuno-enhancer” [50]. Specifically, it has been associated with a reduction in respiratory infection susceptibility in children, and more active youths have been shown to experience fewer sickness days when affected by infections [50,51,52]. Physically inactive children were instead about three times more likely to experience, in a 2-year period, at least one recurrent acute respiratory infection [51]. In adults, regular PA has also been suggested as an additional tool to strengthen the immune system against COVID-19 [53]; however, further studies are needed to draw conclusions in children.

A study involving 88 Chinese children showed the consequences of the polymorphism of the adipokine vistatin on metabolic changes. The association between vistatin and the effect of an aerobic training scheme (performed four times a week for four weeks) was evaluated. There was a considerable reduction in the triacylglycerol levels and insulin sensitivity in subjects that had the polymorphism of vistatin [54].

Another study demonstrated a 92% reduction in the IL-6 concentration and oxidative metabolites of myeloperoxidase in 47 obese patients that underwent intermittent cycling training at 80% VO_2max_ [49].

For the well-known hormone leptin, a study evaluated its levels among active children, reporting that the serum levels were three times lower than among sedentary subjects [55], while a Korean study showed a marked reduction along with increased adiponectin levels in obese children between 10 and 12 years practicing aerobic training at a moderate intensity for 12 weeks. In addition, upon the end of training, the serum levels of these adipokines was stable for three months, though the children did not practice any sports [56].

The weight gained due to decreased PA during the pandemic period has been correlated with a rapid decline in metabolic homeostasis [57], leading to increased levels of laboratory markers related to metabolic disorders such as AST, ALT, triglycerides, and LDL [57]. Metabolic unbalances that occurred during the lockdown periods were evaluated in obese children with nonalcoholic fatty liver disease (NAFLD), a common consequence of childhood obesity [58]; these children had higher blood pressure and glycosylated hemoglobin HbA1c levels compared to the non-NAFLD group [57].

The metabolic and hormonal alterations caused by obesity are also associated with cardiovascular risk factors whose outcomes, later in life, can culminate in death. Constant PA can promote, as early as in childhood, positive cardiovascular effects.

Lastly, PA is known to contribute to the daily energy expenditure and to increase the lean body mass, decreasing the fat mass [59,60], the factors preventing obesity development in children [61,62,63,64], and regulating the body composition during growth [62]. Moreover, PA improves the metabolic profile [59] in terms of the blood glucose levels, insulin resistance, and triglycerides (factors related to improved metabolic and cardiorespiratory fitness in obese children) [61,65] and provides a reduction in mortality [62,66]. For these reasons, PA is fundamental in the treatment of childhood obesity [62,67], and increasing activity levels is a core point in addressing the obesity in pediatric patients [67]. Importantly, in order to be efficacious on the metabolic profile, exercise training does not necessarily need to be vigorous [60]. Indeed, recreational programs have been demonstrated efficacious and may limit the initial dropout rate typical of these patients [60].

A study conducted by Park et al. [68] demonstrated the effect of a training mixed scheme (resistance and aerobic program for 12 weeks) on the endothelial functions in children with obesity. Aerobic activity was performed with 30 min of vigorous walking (almost 60% of the heart rate reserve). A circuit training with exercises involving the upper limbs and the lower limbs constituted resistance training. Higher improvements in the three categories of endothelial progenitor cells were shown; PA stimulated an improvement in the endothelial vasodilator capacity that, in turn, increased the systemic blood flow and decreased the strength of ventricular ejection, diminishing the cardiac overload [68].

The 2016 U.S. Report Card on PA for Children and Youth provided a comprehensive evaluation of the PA status and elements influencing exercise practice among children and youths. It suggested the importance of providing more opportunities for children to be active. There were disparities indicating that particular consideration should be given to some parts of the population such as girls, minorities, and subjects from lower socioeconomic status households [69].

## 4. Changes in Exercise Practices during COVID 19 and Obesity

COVID-19 can affect children, with a milder clinical picture with respect to adults [70]. In the pediatric population, the most common symptoms are mild respiratory symptoms, such as fever, cough, and rhinorrhea [70,71]. Although the majority of pediatric patients have asymptomatic or mild disease symptoms, around 3% develop a severe infection [72], manifesting with dyspnea, cyanosis, low oxygen levels, and acute respiratory distress/failure [72,73]. It was recently reported that children with previous comorbidities have as increased risk of severe COVID-19 manifestations and increased mortality compared to healthy children [74]. Among the comorbidities cited, childhood obesity was related to worse COVID-19 clinical scenarios and was also the most significant factor correlated to the need of mechanical ventilation in the pediatric population [75].

Besides a severe respiratory COVID-19 infection, a new condition has been reported in this virus in children: the so called multisystem inflammatory syndrome in children (MIS-C) [76,77]. This syndrome is considered a postinfectious disease [76,77], although the exact mechanism by which this exaggerated immune response is triggered by SARS-CoV-2 is unknown.

Importantly, during the COVID-19 pandemic, in order to decrease the rapid viral spread, social isolation and lockdown measures were imposed [78]. This dramatically affected the life of children and adolescents, modifying their daily routines, rhythms, and eating behaviors [59,79,80,81,82,83]. Moreover, closures of schools, parks, and gyms and cancellations of organized sports negatively impacted children’s PA and increased their SB [59,79,82,83,84,85]. These restrictive measured were correlated to an increased risk of weight gain, especially in obese children [59,86], and obesity development [59,79,80,87,88]. This phenomenon was shown worldwide [4,80,81,84,89,90,91,92,93]. For instance, in Canada, a decrease in PA level was referred through family administered questionnaires: children were less active, played outside less, and were generally more sedentary, participating in more recreational screen-based activities [81,94]. Moreover, gender and age group-related differences were observed: girls were less active than boys, and adolescents (12–17 years) were more sedentary than younger children (5–11 years) [94].

Interestingly, different factors supporting PA instead of SB were found, such as living in a detached house or having a dog [81]. Among these factors, coherent with previous studies [95,96], the strongest association noted was the parental encouragement for and co-participation in physical movements [81].

A decline in PA was also reported in adolescents and young adults in Spain [97]; overall, Spanish students reduced MVPA during the lockdown period and increased the sedentary time. However, an increase in time spent on “high-intensity interval training” and mind–body activities, such as yoga and Pilates, was reported [97]. This is extremely relevant, as it highlights the possibility of adaptation of PA to confinement measures, a useful way out if future lockdowns or restrictive measures are reiterated [97].

A decrease in PA during the pandemic lockdown was also reported among adolescents in Poland [83] and children in Slovenia [93], Portugal [91], France [92], and the United States [98]. Sport activities also declined in Germany among boys and girls of all age groups [84], and stronger negative effects of the lockdown measures were highlighted among German adolescences, who showed a larger decline of sport activities with respect to younger children [84]. Coherently with the studies cited, Italian children also performed lower levels of PA during the COVID-19 lockdown [59,79,80,82,99], and this led to a vicious circle of SB, decreased energy expenditure, and weight gain [59,100].

Additionally, the results from Latin America, Brazil, and Chile confirmed a decreased PA among adolescents during quarantine and an increase in weight [90].

The lockdown measures thus favored the development of an obesogenic environment [57,80,101], congruously with previous studies reporting a children tend to gain more weight during summer vacations than during school frequency [102,103,104], where structured and constant PA is provided [101,105,106]. This obesogenic environment is harmful both for normal weight children and for obese individuals, to which special attention should be paid [4,59,107], as they already have a higher tendency to engage SB with respect to nonobese children [87]. An interesting microsimulation model was developed in order to evaluate the impact of COVID-19 pandemic on childhood obesity in the United States [90], and it predicted a possible increase in the rate of childhood obesity by 0.64% in children of kindergarten age upon an only 2-month school closure [108].

More in detail, an increase in the BMI in children during the COVID-19 pandemic period was reported in many countries, such as China [109], Palestine [110,111], Italy [59,80], Poland [112], and the USA [113]. Interestingly, the highest increase in the rate of BMI change in children already most vulnerable to unhealthy weight gain (such as children with pre-existing obesity) was found [113], along with the finding that children and adolescents previously inactive or only moderately active increased their sedentariness during lockdown more than active children [80].

Moreover, during the pandemic period, children and adolescents experienced a worsening of their psychological state, documented by increased levels of anxiety, stress, and depressive symptoms [114,115], further worsened by a state of obesity [116]. These depressive symptoms may be also enhanced by a decreased PA, well-known to have a positive influence on the mental health of children and adolescents [117,118,119,120].

In addition to the negative consequences reported at the mental level, a decrease in PA, regardless of the increase in terms of weight, might also have deleterious effects at the physiological level [62,81,88].

These challenging times, although, make it more and more difficult for children—in particular, for obese children already more noncompliant [21,87]—to cope with the recommended activity levels [62,67].

The restrictive measures adopted are extremely relevant for the reduction of the COVID-19 spread [78,121], so new strategies, compatible with lockdown periods, must be found to increase the PA levels in these children [59]. In light of this, a possible tool, already shown to be effective [4,60,97,122] and applied both to healthy children and to children affected by different pathologies [123,124], including obesity [60,125], is the practice of physical exercise via online channels, even in the form of interactive video games [60,126,127].

## 5. Online Exercise: Benefits and Efficacy

Some authors have suggested that, in children and adolescents, the use of video game elements in the home environment contributes to improving the BMI and body weight [128], dietary behavior and nutritional knowledge [129], level of PA, daily caloric expenditure, self-esteem, interactions, and social support [130]. The using of tele-exercise and exergames spread ahead the COVID-19 outbreak, especially among children with pathologies such as cerebral palsy, bone cancer, and cystic fibrosis. This practice aimed to limit public exposure, risks of infections, and transport barriers [131,132,133]. In research conducted in children with cerebral palsy, based on an online individualized exercise program, specialists concluded that the virtual environment was effective in ameliorating the cognitive function thanks to increased levels of attention and concentration [131]. Moreover, other studies in children with spastic hemiplegic cerebral palsy found gains of motor function in the upper limbs and an improvement of manual strength [131]. Chen et al. [132] equipped an interactive online exercise training with wearable devices in children with cystic fibrosis, finding that the streaming of the sessions is a practicable and suitable way to encourage PA practice without cross-infection risks related to in-person group activity. Finally, Cosano et al. [133] showed improvements on bone health in pediatric cancer survivors after an adapted and supervised online exercise program.

On another side point, exergaming is constituted of specific videogames and devices that copy the body movements commuting into the avatar’s moving on screen [134], providing an immersive experience in a three-dimensional realistic setting or a nonrealistic practice with no feeling of being immersed in the virtual world [134]. In this context, as well as in a home-based setting, PA is commuted into the experience of game activities, promoting physical fitness and long-term adherence to training [135]. In addition, exergames were usually perceived as easy to set up and use and shown to be secure for daily use [135]. These features are crucial to ameliorate motivation and self-efficacy and are also easy game requests of movement techniques or coordination. With these configurations, exergames are able to allow sedentary people to approach training and exercises [135].

In children with obesity, exergames have been also offered to ameliorate SB, improving sports engagement and lowering the risk of developing respiratory and cardiovascular pathologies, so as to ultimately increase PA levels [136]. Active videogames should be enjoyable, energizing, and challenging, requesting diversified tasks [137,138]. Research showed that the association of tasks with enjoyment and play time increased the attendance of interventions. The ludic nature of exergaming permitted them to go beyond purely physical and biological features and ascribed a meaning to the playing [137], associating pleasure with health promotion and concurring to limit childhood obesity [139].

Systematic reviews and meta-analyses have indicated that exergames players can perform PA at a light-to-moderate intensity and, during specific tasks or plays, even vigorous intensity, therefore increasing the energy expenditure. In 2013, Lamboglia et al. [140] analyzed, in a systematic review, the use of exergaming to contrast childhood obesity. Exergaming was found to raise the PA levels, energy expenditure, maximal oxygen uptake, and heart rate and to decrease the waist circumference and sedentary screentime. These findings were supportive for children to be more active, especially those who experience strains or discomfort during exercise in public, as children with obesity [141].

Other studies suggested that exergame programs have concrete effects on children’s obesity-related consequences, body composition, and PA attendance. For example, Bethea and colleagues [142] reported an enhancement cardiovascular fitness of children after a 30-week tele-exercise participation. Calcaterra et al. [60] also reported enhancement of the body composition, cardiorespiratory fitness, and metabolic profile after a recreational and supervised 12-week exercise program for sedentary children with obesity, including exergames both during supervised sessions and in a home-based setting. Other researchers found that the effects of an exergame intervention was related to the PA level, BMI, and body fat reduction with specific exergames playing (e.g., Eyetoy and Kinect Sport). In a study with overweight children, Murphy et al. [143] found that the tele-exercise group had a significant improvement in the total exercise time and VO_2_ peak and a reduction of body weight. In addition, from the findings of Daley [144], exergaming increases the energy consumption during leisure time, making children more active and replacing sedentary moments with more active periods. Differently from conventional video games, exergames require full-body participation in different ways [145]. Playing at home, involving the whole family, is crucial for the fight against childhood obesity.

However, several studies have reported that exergame program participation had no effect on children’s BMI and percent of body fat and PA levels [146,147] due to several limitations, such as the total duration of the studies, high dropout rates, and the lack of supervision. Despite this, exergaming technology provides a different framework in which everything and everyone is faster and more engaging, and the chances of winning are greater. Furthermore, children can be involved in sport practicing with actual excitement, inserted into an arena surrounded by cheering crowds, overcoming limits or breaking records, and simulating a sports award ceremony [137]. All these activities could be shared with the whole family, raising the awareness of creating a more active environment in a home-based context. For these reasons, in accordance with Sinclair et al. [148] and Vaghetti et al. [149], exergaming may be considered an attractive, entertaining, and efficient way of engaging in PA while gaining fitness and improving motor skills.

## 6. Online Exercise during COVID-19

During the COVID-19 emergency, WHO promoted the campaign #HealthyAtHome to reinforce the recommendation that children should be healthy and physical active also during the COVID-19 outbreak [150,151]. At the same time, national government dispositions, such as the Italian Government’s #ImStayingHome decree [152], changed the habits and lifestyles of children and adolescents by imposing the closure of schools, isolation at home, and social distancing. In Italy, some advice was provided by the government to invite adults [153] and young people [154] to stay active. Even scientific research sustained the importance of PA and exercise during the COVID-19 emergency under circumstances of sufficient safety [155] and the supervision of trained specialists [156] to avoid physical injuries while home exercising, which is often done incorrectly [157].

To counteract the negatives effects of the restrictive measures adopted during the COVID-19 emergency, some new learning approaches and methodologies were introduced [158,159], i.e., web home-based PA, active video gaming, and physical education, in online learning [6,159]. In this respect, the “gamification” phenomenon is defined as the use of video game elements in nongaming systems to improve user experiences and user engagement [160]. These ones have a strong influence on children and adolescent lifestyles, and they are increasingly employed in learning environments as a way to enhance motivation and encourage social interaction in young people and all family members [161], particularly during the pandemic era. Paradoxically, using a device every day during the pandemic era—not only for exercise but also for online learning and other activities (i.e., calls, video games, chats, or videos)—could cause addiction, and children/adolescents could become active (hypokinetic). For this reason, there is an increased risk of suffering from related psychological factors linked to noncommunicable diseases [162]. Particularly, some authors [163] underlined that overweight children are more incline than their normal weight peers to feel greater psychological distress, low body self-esteem, and to develop depression [164] before and during the COVID-19 lockdown. Stress is notoriously linked with some physiological alterations in overweight people, i.e., the leptin levels are lower, and it also modifies appetite regulation [165].

Many web-based and social media lifestyle suggestions were ideated by private people and professionals (i.e., personal trainers and exercise specialists) [166]. Furthermore, healthy lifestyle platforms were provided by sports federations and public health authorities. For instance, several Italian universities have organized video classes for home-based exercises for young adults [167] with the support of Sport Sciences graduates [166].

To the best of our knowledge, although some experiences have yet to be described for both healthy [168,169,170] and pathological [171,172,173,174,175] adults, the structured online exercise experience for children are limited. Calcaterra et al. [79] proposed an online exercise training intervention through the online platform “LAMAJunior” supported by sport science specialists to counteract a sedentary lifestyle in children. Weiss et al. [176] promoted “Girls on the Run”, an effective program to promote PA and psychosocial well-being during the COVID-19 emergency.

In 2020, the American College of Sports Medicine (ACSM) published some handouts [177] with advice on active gaming and other web resources to render children physically active during the COVID-19 outbreak. More in detail, specific resources were provided as follows. At https://cosmickids.com [178] (accessed on 9 November 2021), some videos of yoga and other mindfulness and relaxation contents were provided with the aim—as stated by the authors—“to help kids build their mental and physical strength and confidence”. Other resources about active videos for indoor activities were provided by GoNoodle^®^ [179], proposing online lessons for primary schoolchildren to enhance personal and community wellness and mental and physical health. The Online Physical Education Network [180], an American public service organization, has made available a series of tools (videos, guides, files, etc.) to maintain physical activity during the COVID-19 emergency. In particular, two specific sections were online, among others: “Active Classrooms” addressed to teachers and “Active Home” addressed to parents and teachers to make the home an active setting and to help both teachers and parents to collaborate together so as to provide more movement opportunities. The American Heart Association (AHA) promoted “School at Home with the AHA” [181], giving online advice and resources about movement and healthy nutrition for families and adults [182].

Many authors have been involved in studying physical education (PE) for online learning during the COVID-19 pandemic, pointing out the critical aspects. In their study, Yu et al. [183] analyzed the effectiveness of online practical classes of PE during the COVID-19 pandemic. The authors emphasized how teachers are required to provide timely and quality feedback, and the students need to be continuously motivated due to the distance and screen mode (rather than face-to-face). In another study, Jeong & So. [184] reported the monotony of the classes within their limited environmental conditions as a critical aspect. Some authors recognized the potential of online PE and the need for adequate preparation of teachers in preparing and conducting practical PE classes online [184,185].

It was also noted how school closures due to COVID-19 created inequity for school-aged children, owing to unequal access to technology and high-speed internet connection, adult support, and physical space to participate in online PE [186]. D’Agostino et al. [186] underlined how inequalities are presented for young people with disabilities. These youngsters are, in fact, particularly dependent on school PE for PA engagement, and therefore, they face barriers in performing at-home PA.

Across the years many studies investigated different approaches and technologies to improve children health through online training, we summarized them in Table 2.

**Figure 1:**
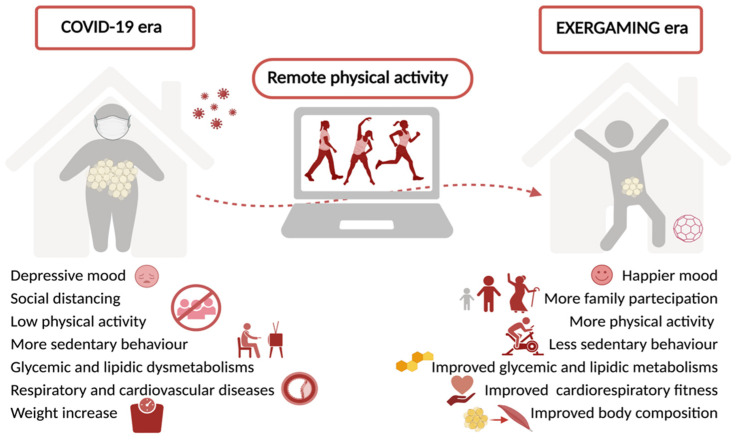
Beneficial effects of remote physical activity and exergaming during COVID-19.

Finally, the benefits of online exercise during the COVID-19 era are shown in Figure 1.

## 7. Concluding Remarks

Obesity is a pandemic emergency affecting people worldwide, and it is even exacerbated by the pandemic infection due to SARS-CoV-2 infection. In a vicious circle, the enacted measures against COVID-19 have augmented sedentary lifestyles, which are detrimental from multiple standpoints. Children and adolescents with obesity are particularly vulnerable to this situation. Having said that, PA and exercise come to the forefront in the battle against pandemics like COVID-19 and obesity. There are a variety of promising approaches to persuade people to keep active, even under the circumstance of home confinement. As a narrative review, this manuscript takes a less formal methodologic approach than systematic reviews. However, it presents an overview on delivering telehealth exercise programs that represent an incredible opportunity for pediatric subjects with obesity to stay healthy, fit, and entertained. Assisting overweight and obese children with remote PA programs may be relevant not only in the case of intermittent lockdowns due to pandemics, but it can also be a valuable strategy for fostering compliance with PA guidelines. These telehealth activities can be conducted either for outdoor or home-based training. Government directives and healthcare policies should exploit this unprecedent scenario as a treasured lesson for implementing insightful interventions. The ultimate aim is to harness the pleiotropic actions of exercise on a regular basis under multiform modalities against comorbidities related to excessive body weight and sedentariness. 

## Figures and Tables

**Table 1:** Diagnostic criteria to classify obesity according to the World Health Organization.

	WHO 2006	WHO 2006	WHO 2007
Age	0–2 years	2–5 years	5–18 years
Index	Weight-to-length ratio	BMI	BMI
>97th percentile	Overweight	Overweight	Obesity
>99th percentile	Obesity	Obesity	Severe obesity

**Table 2:** Studies on online training pre- and during the COVID-19 era.

Authors (Year)	Country	Population	Approach	Resources	Main Findings
Johnson et al. (2018) [131]	Australia	Children with cerebral palsy	Trial	Online individualized exercise program with supervision (16 weeks)	Ameliorated cognitive functions; increased motor function in upper limbs.
Chen JJ et al. (2018) [133]	USA	Children with cystic fibrosis	Trial	Online exercise training with wearable devices (16 weeks)	Increased adherence to PA practice without cross-infection risks
Cosano et al. (2020) [132]	Spain	Children with cancer	Study Protocol	Supervised online exercise program (12 weeks)	Improved on bone health
Chen H et al. (2017) [136]	USA	Children with obesity	Trial	Exergames	Reduced the sedentary behaviors; improved sport engagement and reduced risk to develop respiratory and cardiovascular pathologies.
Lamboglia et al. (2013) [140]	Brazil	Children with obesity	Review	Exergames	Contrasted the childhood obesity rising PA levels, energy expenditure, maximal oxygen uptake, heart rate, and decreasing waist circumference.
Bethea et al. (2012) [142]	USA	Children with obesity	Trial	Tele-exercise (30 weeks)	Enhanced cardiovascular fitness
Calcaterra et al. (2013) [60]	Italy	Children with obesity	Trial	Exergames both with supervision and without (12 weeks)	Enhanced of body composition, cardiorespiratory fitness, and metabolic profile.
Murphy et al. (2019) [143]	UK	Children with obesity	Review	Tele-exercise	Improved total exercise time and VO_2_ peak, with a reduction of body weight.
Daley et al. (2009) [144]	UK	Children with obesity	Review	Exergames (12 weeks)	Increased energy consumption during leisure time.
WHO Campaign #HealthyatHome (2020) [150]	Worldwide	Children (aged 5 to 17 years old)	Campaign	Resources posted online such as video and channels	No data available
Calcaterra et al. (2021) [79]	Italy	Children with type 1 diabetes (aged 5 to 17 years old	Trial	“LAMAJunior” channels with online exercise training	Reduced sedentary habits with shorter beaks of PA practice during the day
Weiss et al. (2021) [176]	USA	Children and young adults	Trial	“Girls on the Run”	PA online program to promote active lifestyle and psychosocial well-being
ACSM Campaign (2020) [177]	USA	Children and young adults	Campaign	Active gaming and other web resources	To increase active lifestyle
Cosmickids Campaign (2021) [178]	No available	Children	Campaign	Yoga and Mindfulness video	To help kids build their mental and physical strength and confidence
GoNoodle (2021) [179]	USA	Primary school children	Campaign	Online lessons	To enhance personal and community wellness and mental and physical health
School at Home with the AHA (2020) [181]	USA	Children and young adults	Campaign	Online advice and resources	To promote an active lifestyle

## Data Availability

Not applicable.
